# A rare case of Kasabach Merritt Syndrome presenting with an infantile hemangioma: A case report

**DOI:** 10.1016/j.amsu.2022.103557

**Published:** 2022-03-29

**Authors:** Abdullah Alghobaishi, Ahmed Hafez Mousa, Haleema Sami Almonaye, Tasneem Khalid Maghrebi, Abdullah Baothman, Fawaz Al Shareef

**Affiliations:** a Department of Pediatrics, Pediatrics Critical Care Medicine, King Fahad Armed Forces Hospital, Jeddah, Saudi Arabia; b Department of Pediatrics, Pediatrics Neurointervention, King Fahad Armed Forces Hospital, Jeddah, Saudi Arabia; c Department of Medicine and Surgery, Batterjee Medical College, Jeddah, Saudi Arabia; d Department of Pediatrics, Saudi German Hospital, Jeddah, Saudi Arabia; e Department of Pediatrics, Pediatric Hematology and Oncology, Ministry of National Guard-Health Affairs, Jeddah, Saudi Arabia; f Department of Pediatrics, Pediatric Hematology and Oncology, King Saud Bin Abdul-Aziz University for Health Sciences (KSAU-HS), Jeddah, Saudi Arabia; g Department of Pediatrics, Pediatric Hematology and Oncology, King Abdullah International Medical Research Center (KAIMARC), Jeddah, Saudi Arabia

**Keywords:** Kasabach Merritt Syndrome, Infantile hemangioma, Thrombocytopenia, Purpura fulminans, Vincristine, COUNT

## Abstract

**Introduction:**

and importance: Infantile hemangioma, being a benign tumor of the blood vessel, is part of a triad composed of also thrombocytopenia and hypofibrinogenemia as part of Kasabach Merrit Syndrome.

**Case presentation:**

We report the case of a 2 months old female Saudi infant referred due to respiratory distress, thrombocytopenia, and enlarging hemangioma on right upper chest, neck, and lower cheek. Diagnosis of kaposiform hemangioendothelioma complicated by Kasabach Merritt thrombocytopenia was done based on the clinical triad of thrombocytopenia, bleeding tendency, and the presence of a vascular tumor.

**Conclusion:**

Vincristine and CTA embolization are lines of management that showed to be the most efficient in the improvement of the clinical picture of KMS in our patient.

## Highlights

• KMS is a rare syndrome and rapid diagnosis is essential for enhanced prognosis.
• A wide range of life-threatening complications can occur rapidly.
• Utilization of vincristine CTA embolization showed dramatic improvement in patient's symptoms.
• KMS necessitates utilizattion of different treatment modalities and a multi-disciplinary team approach.

## 1 Introduction and importance

An infantile hemangioma is a benign tumor which presents as an abnormal cluster of small blood vessels under the skin [1]. Hemangioma is categorized by rapid proliferation if endothelial cells [2]. Kasabach Merritt Syndrome (KMS) is a life-threatening thrombocytopenic coagulopathy associated with rare vascular tumors which has been first described by Kasabach and Merrit in 1940 [3]. The classic triad of KMS is composed of hemangioma, thrombocytopenia, and hypofibrinogenemia [4]. This work has been written in accordance with SCARE criteria [12].

## 2 Case presentation

**Fig. 1:**
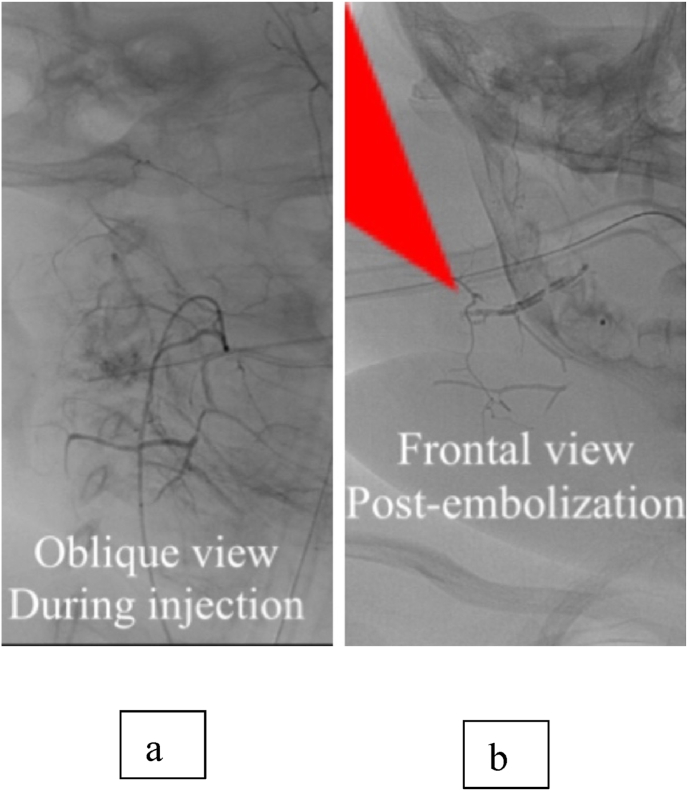
Oblique view during injection (a) and frontal view Post-embolization (b; red arrow). (For interpretation of the references to colour in this figure legend, the reader is referred to the Web version of this article.)

**Fig. 2:**
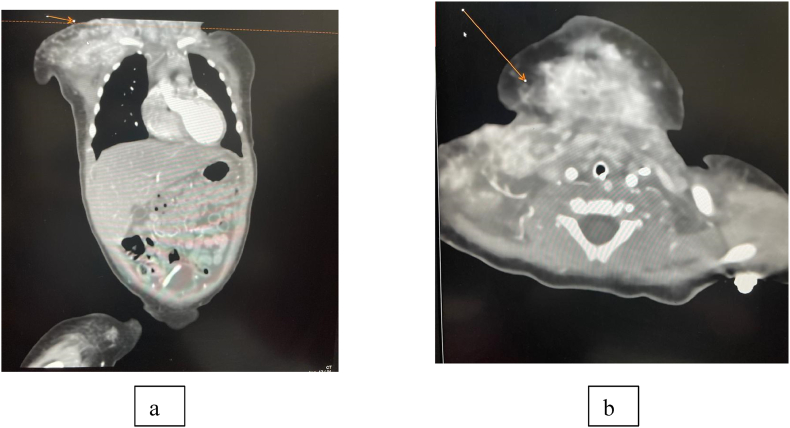
Post-contrast CT of the chest showing an avidly enhanced mass lesion seen in the right chest wall (a) extending to the visualized lower neck and shoulder region with multiple related enhancing vessels representing the feeding arteries and draining veins (b). The orange arrow on both images (a, b) marks the lesion site. There is incidentally noted right sided pneumothorax and left lower lobe patch of consolidation. . (For interpretation of the references to colour in this figure legend, the reader is referred to the Web version of this article.)

This is a 2-month-old Saudi female patient referred from Makkah hospital with the diagnosis of purpuras fulminans, respiratory distress, thrombocytopenia, and enlarging hemangioma on her right upper chest, neck, and lower cheek. She was received by the emergency department, resuscitated, and intubated. The patient had a difficult airway due to that her hemangioma was obstructing her breathing. She received midazolam 2 mcg/kg, and fentanyl 2mcg/kg for sedation. In addition, fresh frozen plasma, cryoprecipitate, and platelets were given to her due to her low platelet level and abnormal coagulation profile. Based on her laboratory findings and clinical features the initial diagnosis was Kasabach Merritt Syndrome. Computed Tomography Angiogram (CTA) of the patient revealed scalp hematoma overlying her right parietal, temporal, and occipital bone with no underlying skull fracture. With further examinations and investigations of the patient, a diagnosis of kaposiform hemangioendothelioma complicated by Kasabach Merritt thrombocytopenia was done based on the clinical triad of thrombocytopenia, bleeding tendency, and the presence of a vascular tumor. Her management included propranolol (0.6 mg/kg/d), methylprednisolone (2mg/kg/d), vancomycin, miropinam, and flagyle, with daily evaluation of platelets and transfusion as needed. Vincrestine, a chemotherapeutic drug was started with a dose of 0.05 mg/kg/week for the desirable effect of hemangioma regression, an effect that was noted a few days later. Computed tomography angiography (CTA) embolization of the external carotid artery was done after stabilization of the patient with complete resolution of hemangioma. Later, she was weaned off steroids, extubated, and started feeding again. However, days later the patient deteriorated with high fever, signs of sepsis, disseminated intravascular coagulation, and rebound of hemangioma with airway obstruction. Initial management done was by intubation, transfusion of platelets, fresh frozen plasma, and cryoprecipitate. She also received vitamin K, meropenem and vancomycin. Her blood cultures revealed the growth of serratia marcescens. She received pulse methylprednisolone and was started again on vincristine with a weekly dose. A second embolization was later done, and the patient was stable again. (Fig. 1) shows micro catheter introducer (Merit MAK™️) femoral access 2.1 F catheter Headway DUO (microvention headway duo microcatheter 156cm) using maximum 4ml/kg contrast, revealed artialized soft tissue tumor supplied mainly by branches of right external carotid artery. Good penetration by 1 ml liquid embolic materials (PHIL) lead to complete obliteration of soft tissue tumor's arterial supply. Residual filling via left ophthalmic artery, no peri procedural complications. (Fig. 2) shows details of the chest wall involvement in addition to an incidental finding of hemangioma.

## 3 Clinical discussion

Kasabach Merritt syndrome is a form of consumptive coagulopathy due to platelet trapping and aggregation within a certain type of hemangioma [5]. The most common site is the skin [6], however, it can grow near vital organs leading to severe disease [7]. Even though it is commonly in countered during infancy [6] there have been reported cases in adults as reported by Liu Y et al. in which a 34 years old female patient with history of multiple giant hepatic hemangiomas during pregnancy and multiple subcutaneous masses which was managed with resection and embolization and was presented to them with petechia, purpura, multiple subcutaneous masses over her limbs and trunk, distended abdomen and hepatomegaly. However, skin manifestations first presented when she was 14 years old [8].

In our case, the patient is a 2-month-old female who presented with a large, extensive, indurated hemangioma on the neck and the right side of the chest with respiratory distress which is differs from the case reported by Morris S et al. in which a male newborn was born with a tumor extending from the mid upper right arm to the wrist associated with consumptive coagulopathy with marked hemodynamic instability as a result of high output heart failure [9]. On the other hand, Abass K et al. reported a case presented with large mass in the abdominal wall [10]. Laboratory evaluation shows variable results of low hemoglobin concentration, low plasma fibrinogen, and prolonged prothrombin time [11]. This child's laboratory tests results showed a hemoglobin level of: 7.8g/dL, platelets: 62,000 μL, prothrombin time: 17.89 sec, partial prothrombin time: 42.73 sec, and D-dimer: 35g/dL which confirmed the diagnosis of consumptive coagulopathy similar to what Liu and Morris et al. reported in their papers [8,9].

Ultrasound (US), computed tomography (CT), and magnetic resonance imaging (MRI) revealed the size, appearance, and layers of hemangioma, its relationships with peripheral blood vessels, and differentiate a hemangioma from vascular malformations [11]. This patient's evaluation with the US of the neck and upper limb was unremarkable as there was no vascular lesion seen and axillary vessels appeared normal. CTA showed a scalp swelling/hematoma overlying right parietal, temporal, and occipital bone, with no underlying skull fracture seen and large *trans*-spatial process extension from anterior chest wall with deep neck extension.

Management of Kasabach Merrit syndrome is very challenging as there are no guidelines yet [10]. Currently, different modalities are recommended including propranolol, steroids, chemotherapy essentially vincristine [5], interferon, sirolimus, embolization, sclerotherapy, radiation, and surgery [8]. In order to manage thrombocytopenia, this patient received multiple plasma transfusions, fresh frozen plasma, and cryoprecipitate. Methylprednisolone 2mg/kg/day was initiated firstly, however, no therapeutic effect was observed and propranolol 0.6 mg/kg/day was added after. Then vincristine 0.05mg/kg/once daily was added. Because all previous measures had been ineffective, CTA embolization under general anesthesia was done and the dose of propranolol increased. Management of this child's case was similar to the management reported by Radović et al. However, management with propranolol and methylprednisolone improved the values of monitored parameters and the child underwent surgical resection of the tumor successfully in the case discussed by Radović et al. [11].

## 4 Conclusions

When evaluating patients with such vascular malformations, and systemic associations should be extensively investigated in order to avoid delay in diagnosis of uncommon syndromes. Variation in response to propranolol among KMS patients across the literature and in our study existed notably. Vincristine and CTA embolization are lines of management that showed to be the most efficient in the improvement of the clinical picture of KMS in our patient.

## Sources of funding

None.

## Ethical approval

Ethical approval has been given by the Institutional Review Board (IRB) of our institution, Saudi German Hospital, Jeddah, Saudi Arabia with the number of H-06-KM-103.

## Consent

This is a single case report with no identifiable patient information are included in this case report. An Institutional Review Board (IRB) approval however was obtained from Saudi German Hospital, Jeddah, Saudi Arabia prior to the conduction of the report.

## Author contributions

• Drafting of the manuscript: Ahmed Hafez Mousa, Haleema Sami Almonaye, Tasneem Khalid Maghrebi
• Critical revision of the manuscript for important intellectual content: Ahmed Hafez Mousa, Haleema Sami Almonaye, Tasneem Khalid Maghrebi, Abdullah Alghobaishi, Abdullah Baothman, Fawaz Al Shareef

## Trial registry number

Name of the registry:
Unique Identifying number or registration ID:
Hyperlink to your specific registration (must be publicly accessible and will be checked):

## Guarantor

Ahmed Hafez Mousa, corresponding author of the manuscript, accept full responsibility for the work and the conduct of the study, had access to the data, and controlled the decision to publish.

## Patient consent

Written informed consent was obtained from the patient for publication of this case report and accompanying images. A copy of the written consent is available for review by the Editor-in-Chief of this journal on request.

## Provenance and peer review

Not commissioned, externally peer reviewed.

## Statement of ethics

This study complies with internationally accepted standards for research practice reporting.

## Declaration of competing interest

None.
